# Is intermediate risk really intermediate? Comparison of karyotype and non‐invasive prenatal testing results of pregnancies at intermediate risk of trisomy 21 on maternal serum screening

**DOI:** 10.1002/jgc4.1973

**Published:** 2024-10-04

**Authors:** Gul Alkan Bulbul, Emine Kirtis, Hulya Kandemir, Cem Yasar Sanhal, Sezin Yakut Uzuner, Sibel Berker Karauzum, Ibrahim Inanc Mendilcioglu

**Affiliations:** ^1^ Division of Perinatology, Department of Gynecology and Obstetrics Akdeniz University Faculty of Medicine Antalya Turkey; ^2^ Department of Medical Biology and Genetics Akdeniz University Faculty of Medicine Antalya Turkey

**Keywords:** intermediate risk, karyotype, non‐invasive prenatal screening, prenatal diagnosis

## Abstract

The purpose of this study was to assess the additional contribution of karyotyping compared with genome‐wide non‐invasive prenatal testing (NIPT) for pregnancies at intermediate risk for trisomy 21 (T21), calculated using the maternal serum screening without major structural anomalies detected through sonography. Karyotype results of all pregnancies undergoing invasive prenatal diagnostic testing between January 2013 and March 2022 were obtained from a large hospital‐based laboratory. Pregnancies with no major structural anomalies on ultrasound (including soft markers) and an intermediate risk for T21 on maternal serum screening were included in this study. The additional contribution of karyotyping for abnormal karyotype results was calculated after excluding results that could theoretically be identified with genome‐wide NIPT. Among the 511 pregnancies analyzed, 13 (2.54%) were found to have abnormal karyotype results, 9 (1.76%) of which could theoretically have been detected with genome‐wide NIPT. Within the cohort, 6/263 (2.28%) of women aged 35 years and older, and 3/248 (1.20%) of women younger than 35 years had results that could have been detected with genome‐wide NIPT. After excluding results detectable using genome‐wide NIPT, the additional contribution of karyotyping was found as 4/502 (0.79%) for the entire cohort, 2/257 (0.77%) for women aged 35 years and older, 2/245 (0.81%) for women younger than 35 years. Of the 511 examined pregnancies at intermediate risk for T21 by maternal serum screening, genome‐wide NIPT would have failed to detect 4 of 13 abnormal karyotype results. The findings hold importance in guiding couples' informed decision‐making processes regarding their choice of genetic screening and diagnostic testing in case of intermediate risk for T21.

**What is known about this topic?**

NIPT is considered the most specific and sensitive screening test for common fetal aneuploidies.

**What this paper adds to the topic?**

In pregnancies at intermediate risk for trisomy 21 without major structural anomalies detected using sonography, low‐risk NIPT results still carry a residual risk of abnormal karyotype results. Determining the rate of abnormal karyotype findings following a low‐risk NIPT outcome holds significant importance for patients, obstetricians, and genetic counselors, enabling them to make well‐informed decisions regarding prenatal testing and screening alternatives.

## 1 INTRODUCTION

In general, chromosomal abnormalities are observed in an incidence of 1 in 150 live births (Nussbaum et al., 2016). Trisomy 21 (T21) is the most common chromosomal anomaly among neonates occurring in 1 of 660 live births worldwide (Jones, 2013). Prenatal screening is performed because T21 is associated with significant intellectual disability and various medical impairments. Prenatal diagnosis allows women options including preparing for the birth of an affected child or termination of pregnancy.

Since the identification of cell‐free DNA (cfDNA) of fetal/placental can be performed in the maternal circulation, NIPT has been successfully used for fetal chromosomal aneuploidy screening such as T21, trisomy 18 (T18), trisomy 13 (T13), and the sex chromosome aneuploidies (SCAs) in clinical practice since 2011 (Agarwal et al., 2013; Lo et al., 1997). NIPT is currently the best non‐invasive method to assess the risk of T 21, T18, and T13 (American College of Obstetricians and Gynecologists’ Committee on Practice Bulletins—Obstetrics; Committee on Genetics; Society for Maternal‐Fetal Medicine, 2020). In a meta‐analysis conducted by Gil et al. (2017) the detection rates were found as 99.7%, 97.9%, and 99.0% for T21, T18, and T13, respectively, and the rate of false‐positive results was 0.04%. Further advances in NIPT technology have shown that rare autosomal trisomies (RATs), recurrent microdeletion/microduplication syndromes, and even genome‐wide copy‐number variants could be detected. An expanded NIPT includes recurrent microdeletions (such as 1p36.3‐1p36.2, 4p16.3‐4p16.2, 5p15.3‐5p15.1, 15q11.2‐15q13.1, and 22q11.1.2 deletions) and genome‐wide NIPT usually detects deletions and duplications greater than 5 Mb (Chen et al., 2021; Hu et al., 2022; Wapner et al., 2015).

To screen for common trisomies in the general population, NIPT can be used as a first‐line screening method or depending on the results of the combined test performed at 11 and 13 weeks of gestation (contingent NIPT screening; Gil et al., 2016). Contingent NIPT screening is a strategy with a much higher detection rate and significantly lower cost compared to the use of NIPT as a first‐line screening method (Kagan et al., 2015). This strategy also offers the advantages of correct gestational age calculation, ultrasound examination for the early detection of several major fetal defects, and first‐trimester biochemical testing (Nicolaides, 2011). Different cut‐off values classified as intermediate risk have been used in contingent NIPT screening (1 in 100 or 150 and 1 in 1000–2500; Chitty et al., 2016; Gil et al., 2016; Miltoft et al., 2018). In a study, Miltoft et al. found that an invasive test offered to women who had risk of ≥1 in 100 in the first‐trimester screening test, and contingent NIPT screening offered to women who had a risk of 1 in 100 to 1 in 1000 had the same sensitivity for T21, T18, and T13 compared with offering an invasive test to all women who had a risk of ≥1 in 300 in the first‐trimester screening test. The false‐positive rate was found as 1.2% in the two‐stage model, whereas it was 3.0% in the conventional approach (Miltoft et al., 2018).

**FIGURE 1:**
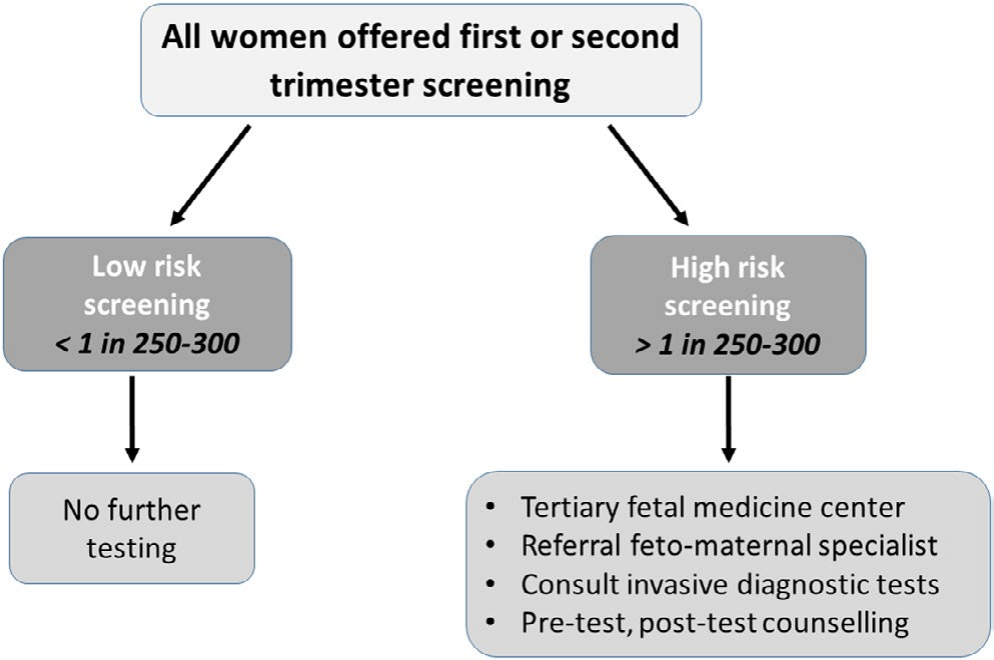
The National Prenatal Screening Strategy For Trisomy 21 in Turkey.

In Turkey, prenatal care is provided both by public and private sectors. In the public health system, prenatal care is free of charge which is predominantly performed by family practice providers and obstetricians. According to the Ministry of Health Prenatal Care and Management Guideline, all pregnant women should receive at least four sessions of prenatal care during their pregnancy, as the first one takes place within 12 weeks of gestation. Additionally, all pregnant women should be routinely offered prenatal screening as a part of their prenatal care (Guide, 2018). Traditional screening tests applied in Turkey are first‐trimester screening (nuchal translucency measurement and measurement of serum analytes) between 11 and 14 weeks of gestation, triple/quad screening (measurement of maternal serum analytes) between 16 and 20 weeks of gestation, and fetal anatomic ultrasound survey between 18 and 22 weeks of gestation. Despite NIPT is currently available through private providers, it is not yet available in publicly funded prenatal services. Pregnancies with abnormal serum screening (at the threshold of 1:250–300) or with sonographic anomalies are referred to the tertiary fetal medicine centers for further invasive prenatal diagnostic testing (chorionic villus sampling, amniocentesis, or cordocentesis). Moreover, the invasive tests are also financed by government in the pregnancies of mothers aged 35 years and older, the parental chromosomal rearrangements, past history of a fetus or infant with T21, and intermediate risk for T21 at the parents' own discretion. Prenatal invasive procedures are generally performed by the fetal–maternal medicine team, while both pre‐ and posttest counselings are done by clinical geneticists. Last but not least, maternal serum screening tests and karyotype analysis are generally performed in in‐house laboratories. The national prenatal screening strategy (current practice) in Turkey is illustrated in Figure 1.

The performance of NIPT in pregnancies at intermediate risk for T 21 has been defined in large cohorts (Gil et al., 2015; Kagan et al., 2015; Nicolaides et al., 2013, 2014); however, there is limited evidence showing the comparative results of NIPT and karyotyping data in this group. In our study, we aimed to assess the additional contribution of karyotyping compared with genome‐wide NIPT for pregnancies at intermediate risk for T21 based on a large karyotyping cohort that had no major structural anomalies.

## 2 MATERIALS AND METHODS

This retrospective cohort study was based on the database of the Center of Genetic Diagnosis, Akdeniz University Hospital (Antalya, Turkey). After obtaining approval from the Akdeniz University Faculty of Medicine Clinical Research Ethics Board (Number: 326, Date: 15.05.2022), we retrospectively assessed the karyotype.

Results of all pregnancies who underwent invasive prenatal diagnostic testing at the outpatient clinic of Perinatology, Department of Obstetrics and Gynecology, Akdeniz University Hospital between January 2013 and March 2022. Eligibility criteria included: (1) pregnancies undergoing diagnostic testing due to intermediate risk for trisomy 21 by maternal serum screening with normal ultrasound and (2) accompanied with or without the soft markers. Pregnancies with multiple fetuses or sonographic anomalies (including structural anomalies, nuchal translucency over 3 mm, polyhydramnios, or intrauterine growth restriction) were excluded from the study.

The risk of T21, calculated using the combined first‐trimester screen, ranging from 1/101 to 1/1000, is classified as intermediate risk as proposed by Nicolaides et al. (Chitty et al., 2016). Demographic characteristics of patients, antenatal screening methods (double, triple, and quadruple test), presence of minor markers in ultrasonography, gestational weeks, and the invasive method technique applied (chorionic villus biopsy, amniocentesis, and cordocentesis) were recorded. The karyotyping results were categorized as normal or abnormal, and abnormal results were further divided into subgroups based on the type of abnormality. Abnormal results were analyzed according to the type of abnormality and whether they could be detected through genome‐wide NIPT.

Non‐mosaic T13, T18, T21, or SCAs, recurrent microdeletions (1p36.3‐1p36.2, 4p16.3‐4p16.2, 5p15.3‐5p15.1, 15q11.2‐15q13.1, and 22q11.1.2 deletions), and deletions and duplications greater than 5 Mb were considered detectable with genome‐wide NIPT (Hu et al., 2022). Deletions and duplications smaller than 5 Mb, as well as any form of mosaicism, were considered non‐detectable with genome‐wide NIPT. The additional contribution of karyotyping for abnormal results was calculated by excluding results that could be detectable via NIPT. Subgroup analysis was performed for pregnancies with an advanced maternal age (i.e., aged 35 years and older) versus younger women. The Chi‐square test with Yates's correction was used to compare the detection rates between subgroups. A P‐value of <0.05 was considered statistically significant.

## 3 RESULTS

**FIGURE 2:**
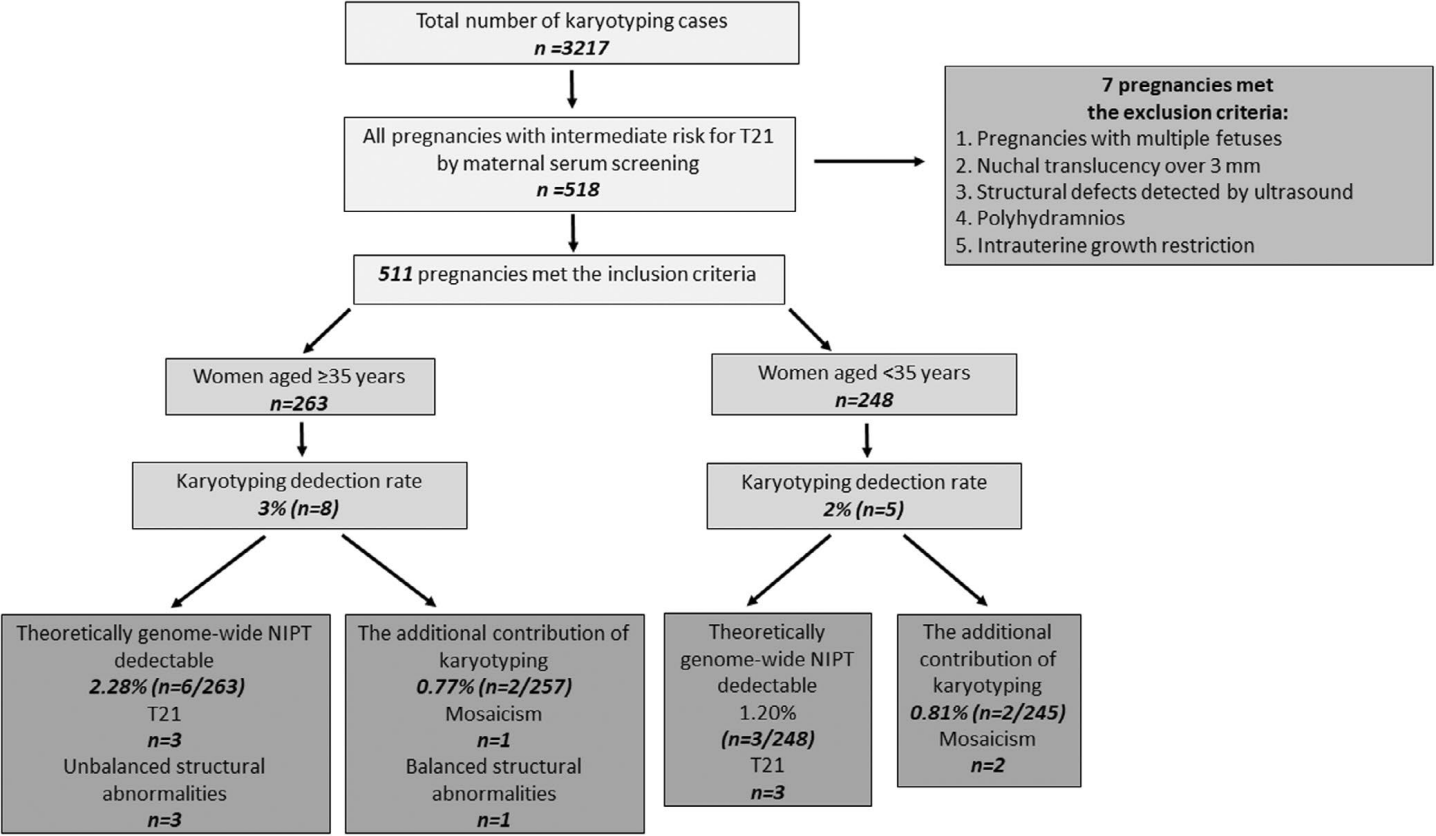
Flow chart describing the karyotyping detection rates in pregnancies with intermediate risk for T21 by maternal serum screening and the contributions of karyotyping after excluding theoretically genome‐wide NIPT detectable findings. NIPT, non‐invasive prenatal test; T21, trisomy 21.

During the study period, karyotype analyses were performed on 3217 prenatal samples in a genetic laboratory. Among them, 518 were found to be pregnancies with intermediate risk for T21 by maternal serum screening. After excluding 7 cases with multiple fetuses and sonographic anomalies (including structural defects, nuchal translucency over 3 mm, polyhydramnios, or intrauterine growth restriction), 511 pregnancies were included in the study. Among 511 analyses, 263 were performed in women aged 35 years and older and 248 in younger than 35 years (Figure 2).

Regarding the prenatal invasive methods used, 500 patients underwent amniocentesis, 7 underwent chorionic villus biopsies, and 4 underwent cordocentesis. The mean maternal age was 33.5 ± 2.7 years. The mean gestational age at CVS, amniocentesis, and cordocentesis was 12.2 ± 5, 18.1 ± 2, and 21.6 ± 1 weeks, respectively. There were no procedure‐related miscarriages among the 511 patients. Soft markers were detected in eight patients, including pyelectasis in five, choroid plexus cyst in two, and a hyperechogenic bowel in one patient.

Of the 511 patients who underwent prenatal invasive procedures, 13 cases (2.5%) were found to have abnormal karyotype results. Among them, 8 (3%) were in women aged 35 years and older, whereas 5 (2%) were in women younger than 35 years. Regarding the distribution of the anomalies, T21 was the most frequent abnormality at 46.1% (6/13), followed by mosaicism at 23% (3/13), unbalanced structural abnormalities (deletion, derivative chromosome, and isochromosome) at 23% (3/13), and balanced structural abnormalities (translocation) at 7.6% (1/13). Table 1 and Figure 2 summarize abnormal karyotype results in the examined cohort.

**TABLE 1:** Abnormal karyotyping results grouped by maternal age.

Maternal age at time‐of‐invasive procedure (years)	Karyotype results	*n*	Detectable with genome‐wide NIPT versus karyotyping
35≥	47,–,+21	3	Detectable with Genome‐wide NIPT
	46,X,i(X)(q10)	1	Detectable with Genome‐wide NIPT
	46,–,del(18p11.23) dn	1	Detectable with Genome‐wide NIPT
	46,–,der(22)t(Y;22)(q12;p11.2) pat	1	Detectable with Genome‐wide NIPT
	46,–,t(1;6)(q21;p23) pat	1	Detectable only by karyotyping
	mos 47,–,+21[26]/46,– [74]	1	Detectable only by karyotyping
35<	47,–,+21	3	Detectable with Genome‐wide NIPT
	mos 92,XXYY [30]/46,XY [70]	1	Detectable only by karyotyping
	mos 46,–,del(10)(q24.1)[6])/46,–[44]dn	1	Detectable only by karyotyping

*Note*: The gray shading demonstrates the karyotype results that are theoretically detectable with Genome‐wide NİPT.

Abbreviations: “–”, undisclosed fetal sex; del, deletion; der, derivative; dn, de novo; i, isochromosome; mos, mosaic; NIPT, non‐invasive prenatal test; pat, paternal; t, translocation.

Theoretically, 9 of 13 abnormal karyotype results (69.2%) could have been detected using genome‐wide NIPT. Among them, six were T21 (47,–,+21) cases, one was Turner syndrome with isochromosome X (46,X,i(X)(q10)), while one was a deletion (46,–,del(18p11.23) dn) case, and one was a derivative chromosome case (46,–,der(22)t(Y;22)(q12;p11.2) pat). The percentage of results detectable using genome‐wide NIPT was 9/511 (1.76%) for the entire cohort, 6/263 (2.28%) for women aged 35 years and older, and 3/248 (1.20%) for women younger than 35 years. When results detectable using genome‐wide NIPT were excluded, the additional contribution of karyotyping was 4/502 (0.79%) for the entire cohort, 2/257 (0.77%) for women aged 35 years and older, and 2/245 (0.81%) for women younger than 35 years. The difference was not significant (*p* > 0.05). Additional contributions of karyotyping are summarized in Table 2.

**TABLE 2:** Additional contribution of karyotyping.

Detection method	Total *n* (%)	Older than 35 *n* (%)	Younger than 35 *n* (%)
Detectable with genome‐wide NIPT	9/511 (1.76%)	6/263 (2.28%)	3/248 (1.20%)
Detectable by karyotyping	13/511 (2.54%)	8/263 (3.04%)	5/248 (0.80%)
Additional contribution of karyotyping	4/502 (0.79%)	2/257 (0.77%)	2/245 (0.81%)

Abbreviation: NIPT, non‐invasive prenatal test.

## 4 DISCUSSION

Our study reveals that genome‐wide NIPT would have missed 4 abnormal karyotype results out of 13 abnormal results, as per the karyotyping results of 511 pregnant women in the intermediate‐risk group for T21 who had no major structural anomalies. After excluding results detectable using genome‐wide NIPT, the additional contribution of karyotyping for abnormal karyotype results was found to be 0.79%. This additional contribution was 0.81% for women younger than 35 years and 0.77% for women aged 35 years and older. Considering the contribution of karyotyping, if NIPT had been used as the first‐line screening method for patients in the intermediate‐risk group, it would have missed three mosaic results (mosaic trisomy 21, mosaic tetraploidy, and mosaic deletion) and one balanced structural anomaly (balanced translocation) although it would have detected six T21 results and three unbalanced structural anomalies (derivative chromosome, deletion, and isochromosome).

Despite low‐risk NIPT results significantly reducing the aneuploidy risk, it does not guarantee a normal karyotype. The notable residual risk was demonstrated in different groups for clinically significant chromosomal microarray analysis (CMA) results after excluding NIPT results. In a study by Maya et al. (2022), the residual risk for clinically significant copy number variants in low‐risk pregnancies with normal serum biochemistry was 0.68% for the entire group, 0.82% for women older than 35 years, and 0.5% for women younger than 35 years after excluding genome‐wide NIPT results. In another study, 21 clinically significant abnormalities were found in CMA among 559 amniocentesis cases performed due to abnormal maternal serum screening results. The residual risk for CMA findings was 2.0% after theoretically normal NIPT results (Sagi‐Dain et al., 2022). In another retrospective cohort study, among 122 clinically significant CMA results from 8605 pregnancies with normal ultrasound results (including abnormal serum biochemistry and soft markers), the additional contribution of CMA following omission of genome‐wide NIPT detectable findings was 0.91% for the entire cohort, 1.01% for women older than 35 years, and 0.61% for women younger than 35, whereas it was 1.74% for pregnancies with abnormal serum biochemistry and 0.95% for the soft marker group (Maya et al., 2023).

The frequency of fetal chromosome abnormality detection depends on clinical indications (Nishiyama et al., 2015). In our cohort, the incidence of chromosomal abnormalities was 2.5% with 61.5% (8/13) of chromosomal abnormalities detected in women aged 35 years and older, and 38.5% (5/13) detected in women younger than 35 years. T21 (46.6%) was the most frequent trisomy for both age groups. These results appear to be consistent with the previous studies reporting that the incidence of chromosomal anomalies and T21 ranged from 2.9% to 3.6% and 36.9% to 61.9%, respectively (Han et al., 2008; Mademont‐Soler et al., 2011; Ocak et al., 2014). This finding may be significant when offering prenatal genetic counseling to patients in the intermediate‐risk group for T21.

Fetal mosaicism was detected in three of the abnormal karyotype results in our study (mosaic trisomy 21, mosaic tetraploidy, and mosaic deletion). Postzygotic mitotic errors lead to chromosomal mosaicism in approximately 1–2% of chorion villus samples (Hahnemann & Vejerslev, 1997). The source of cell‐free DNA is apoptotic cytotrophoblasts secreted from placental chorionic villi to maternal blood. The cytotrophoblast layer of chorionic villi does not always represent the fetus because it is derived from the trophoblasts of blastocysts, whereas the fetus comes from the inner cell mass. Cytotrophoblasts, mesenchymal nuclei, and fetal karyotypes may differ (Bianchi et al., 1993). If abnormal cells are limited to the cytotrophoblast layer, they are classified as confined placental mosaicism (CPM) type 1. In CPM type 2, abnormal cells are limited to the mesenchyme. In CPM type 3, abnormal cells are present on both layers of the chorionic villi but absent in the fetus. True fetal mosaicism is associated with a normal karyotype in cytotrophoblasts while the fetus itself has an abnormal karyotype and is classified as true fetal mosaicism type 5 (TFM 5; Grati et al., 2014). In a review, it was reported that CPM was the cause of 32% of all false‐positive cases in NIPT, and TFM type 5 was the cause of 92% of false‐negative cases (Hartwig et al., 2017). Due to limited reports on mosaic results detectable via NIPT, the accurate detection rate remains unclear (Brison et al., 2018). In our study, we also considered mosaic results as detectable only by karyotyping.

In our study, balanced structural anomaly (balanced translocation) was detected in one of the abnormal karyotype results. Balanced translocations are chromosomal rearrangements consisting of components where two different chromosomes change segments without genetic material gain or loss, with a frequency of 0.08%–0.3% in the general distribution (Zhang et al., 2016). In patients carrying balanced reciprocal translocations, mispairing of translocated chromosomes during the first meiotic division can lead to different forms of segregation, which can result in balanced, unbalanced, normal gametes of the translocated chromosomes and may cause the transmission of chromosomal abnormalities to their offspring (Pourjafari et al., 2012). CMA is recommended as the first‐line test in cases of fetal structural anomalies and/or stillbirth, and it replaces the need for karyotyping in most cases. However, it is not able to identify balanced translocations or inversions. Karyotyping remains an important method in prenatal diagnosis of balanced translocations (Sparks & Dugoff, 2023).

In prenatal series, the estimated prevalence of microdeletions included in extended NIPT is 1: 5000 (such as 1p36.3‐1p36.2, 4p16.3‐4p16.2, 5p15.3‐5p15.1, 15q11.2‐15q13.1, and 22q11.1.2 deletions; Srebniak et al., 2018). Although the majority of pathogenic microdeletion/ duplication syndromes cause miscarriage in the first trimester of pregnancy or present as abnormal ultrasound findings during routine developmental checks, some are potentially viable to term and result in developmental and/or physical disorders (Chen et al., 2017). No cases of microdeletion/microduplication were detected in our study. This may be due to the inclusion of pregnancies with normal ultrasound and the small sample size in our study. Therefore, microdeletion/duplication screening in our cohort would not have altered results.

In our study, there were no procedure‐related miscarriages among the 511 patients. A meta‐analysis performed in 2019 suggested that the procedure‐related risk of pregnancy loss after amniocentesis and CVS was lower than the risk currently quoted to women and it might be 1/300 at the most (Salomon et al., 2019). There is evidence highlighting the fact that the risk of miscarriage from invasive procedures is related to the skill and experience of the operator (Bakker et al., 2017). The fact that the results of our study were obtained from a specialized fetal medicine center and thus all procedures in the study were either undertaken or directly supervised by fetal medicine specialists may have contributed to the avoidance of fetal loss.

Indeed, both the American College of Obstetricians and Gynecologists (ACOG) and the American College of Medical Genetics and Genomics (ACMG) emphasize the importance of both pre‐ and posttest counseling as essential components of prenatal genetic testing (American College of Obstetricians and Gynecologists’ Committee on Practice Bulletins—Obstetrics; Committee on Genetics; Society for Maternal‐Fetal Medicine, 2020; Dungan et al., 2023). The purpose of pre‐ and postgenetic test counseling is to ensure patient‐informed consent and autonomous decision‐making (Rink & Kuller, 2018). In fact, in Turkey, no standard procedure is currently applicable for genetic counseling regarding prenatal screening and diagnostic testing for T21 during antenatal care. The primary obstetrical provider (obstetrician and family practice provider) is the initial source of information for patients about genetic testing. There are no special training and/or certificate programs for genetic counseling by non‐physicians. Clinical geneticists (with the addition of a medical genetic residency or pediatric genetic fellowship training after pediatric residency) are employed in the tertiary genetic centers as ‘Genetic Counselors’. Furthermore, most pregnant women in Turkey meet genetic counselors, when they are diagnosed as “high‐risk for T21.” This timing is too late to counsel couples about prenatal genetic screening and diagnostic testing. Studies from Turkey affirm that women do not have enough knowledge to be able to make an informed decision concerning prenatal screening and diagnostic testing (Seven et al., 2017, 2019). In fact, there are no national guidelines/policies for NIPT in Turkey. Self‐financed NIPT is offered by obstetricians as either a first‐line screening test or pregnancies at intermediate risk of T21 after first‐ or second‐trimester screening test results through private clinics and NIPT performance does not require genetic counseling. Therefore, the couples are not exposed to the information and understanding of the limitations of NIPT which potentially compromises the couple's autonomy in prenatal decisions.

There are several limitations of this study. First, the study cohort is biased selectively due to the retrospective analysis of pregnancies at intermediate risk of T21 that had undergone diagnostic testing. Second, the specific maternal serum screening risk was not determined through a standard test but through first‐trimester tests, triple tests, and quadruple tests. Another limitation was the lack of CMA results for pregnant women at intermediate risk for T21 which could detect critical anomalies not detected in karyotyping and could impact neonates in the future. The theoretical definition of NIPT‐detectable findings may be inaccurate because this technique has false‐negative and false‐positive rates. In addition, some pregnancies with NIPT‐detectable aberrations may result in miscarriage prior to amniocentesis. Unfortunately, data on later prenatal and postnatal follow‐up were not available. Finally, additional factors could potentially influence the residual risk such as ethnicity, maternal weight, or paternal age. Given these limitations and considering the biases of the cohort with diagnostic testing, the results may not be generalizable to general population at intermediate risk for T21. A randomized clinical trial in a larger cohort is needed to objectively evaluate the additional contribution of karyotyping in pregnancies at intermediate risk for T21.

Nevertheless, our results show that in pregnancies at intermediate risk for T21 without major structural anomalies detected in sonography, low‐risk NIPT results still carry a residual risk of chromosomal abnormalities detected by karyotyping. This knowledge is important for the patients, obstetricians, and genetic counselors to facilitate informed decisions regarding prenatal testing and screening options.

## AUTHOR CONTRIBUTIONS

Gul Alkan Bulbul agrees to be responsible for investigating and resolving questions related to the accuracy and integrity of the article data in a suitable manner and to make significant contributions to the understanding and design of the study. She has contributed to obtaining, analyzing, and interpreting the data for the project. Emine Kirtis prepared the draft of the study and critically reviewed it for significant intellectual content. Hulya Kandemir conducted the data acquisition, analysis, and interpretation for the study. Cem Yasar Sanhal and Ibrahim Inanc Mendilcioglu prepared the draft of the study and critically reviewed it for significant intellectual content, providing final approval for the published version. Sezin Yakut Uzuner and Sibel Berker Karauzum made significant contributions to the understanding or design of the study, as well as to data acquisition, analysis, interpretation, and final approval of the published version.

## FUNDING INFORMATION

The authors declare that they have not received any financial support for this study.

## CONFLICT OF INTEREST STATEMENT

The authors declare that they have no potential conflict of interest regarding the investigation, authorship, and/or publication of this article.

## ETHICS STATEMENT

Human studies and informed consent: This study was reviewed and approved by Akdeniz University Faculty of Medicine Clinical Research Ethics Board with Approval No: 326, dated 15.05.2022. The rights of all patients were protected, and written informed consent was obtained prior to the procedures in compliance with the Helsinki Declaration.

Animal studies: No non‐human animal studies were carried out by the authors of this article.

## Data Availability

The data that support the findings of this study are available on request from the corresponding author.
